# The Non-Steroidal FXR Agonist Cilofexor Improves Portal Hypertension and Reduces Hepatic Fibrosis in a Rat NASH Model

**DOI:** 10.3390/biomedicines9010060

**Published:** 2021-01-09

**Authors:** Philipp Schwabl, Eva Hambruch, Grant R. Budas, Paul Supper, Michael Burnet, John T. Liles, Manfred Birkel, Ksenia Brusilovskaya, Philipp Königshofer, Markus Peck-Radosavljevic, William J. Watkins, Michael Trauner, David G. Breckenridge, Claus Kremoser, Thomas Reiberger

**Affiliations:** 1 Division of Gastroenterology and Hepatology, Department of Internal Medicine III, Medical University of Vienna, 1090 Vienna, Austria; philipp.schwabl@meduniwien.ac.at (P.S.); paul.supper@meduniwien.ac.at (P.S.); ksenia.brusilovskaya@meduniwien.ac.at (K.B.); philipp.koenigshofer@meduniwien.ac.at (P.K.); markus@peck.at (M.P.-R.); michael.trauner@meduniwien.ac.at (M.T.); 2 Vienna Hepatic Experimental Hemodynamic (HEPEX) Laboratory, Medical University of Vienna, 1090 Vienna, Austria; 3 Christian Doppler Lab for Portal Hypertension and Liver Fibrosis, Medical University of Vienna, 1090 Vienna, Austria; 4 Phenex Pharmaceuticals AG, 69123 Heidelberg, Germany; eva.hambruch@phenex-pharma.com (E.H.); manfred.birkel@phenex-pharma.com (M.B.); claus.kremoser@phenex-pharma.com (C.K.); 5 Gilead Sciences Inc., Foster City, CA 94404, USA; grant.budas@gilead.com (G.R.B.); john.liles@gilead.com (J.T.L.); will.watkins@gilead.com (W.J.W.); david.breckenridge@gilead.com (D.G.B.); 6 Synovo GmbH, 72076 Tübingen, Germany; michael.burnet@synovo.com; 7 Department of Internal Medicine and Gastroenterology (IMuG), Hepatology, Endocrinology, Rheumatology, and Nephrology with Centralized Emergency Service (ZAE), Klinikum Klagenfurt am Wörthersee, 9020 Klagenfurt, Austria; 8 Ludwig Boltzmann Institute for Rare and Undiagnosed Diseases (LBI-RUD), 1090 Vienna, Austria; 9 CeMM Research Center for Molecular Medicine, The Austrian Academy of Sciences, 1090 Vienna, Austria

**Keywords:** NASH, FXR, cilofexor, portal hypertension, fibrosis, rats, NAFLD, farnesoid X receptor, cirrhosis, propranolol

## Abstract

Background: The farnesoid X receptor (FXR) influences hepatic metabolism, inflammation and liver fibrosis as key components of non-alcoholic steatohepatitis (NASH). We studied the effects of the non-steroidal FXR agonist cilofexor (formerly GS-9674) on portal pressure and fibrosis in experimental NASH. Methods: NASH was induced in Wistar rats using a choline-deficient high-fat diet plus intraperitoneal sodium nitrite injections. First, a dose-finding study was performed with 10 mg/kg and 30 mg/kg of cilofexor, focusing on histological readouts. Liver fibrosis was assessed by Picro-Sirius-Red, desmin staining and hepatic hydroxyproline content. Gene expression was determined by RT-PCR. In a subsequent hemodynamic study, rats received 30 mg/kg cilofexor with or without propranolol (25 mg/kg). Portal pressure, systemic hemodynamics and splanchnic blood flow were measured. Results: Cilofexor dose-dependently induced FXR target genes *shp, cyp7a1* and *fgf15* in hepatic and ileal tissues, paralleled by a dose-dependent reduction in liver fibrosis area (Picro-Sirius-Red) of −41% (10 mg/kg) and −69% (30 mg/kg), respectively. The 30 mg/kg cilofexor dose significantly reduced hepatic hydroxyproline content (−41%), expression of *col1a1* (−37%) and *pdgfr-β* (−36%), as well as desmin area (−42%) in NASH rats. Importantly, cilofexor decreased portal pressure (11.9 ± 2.1 vs. 8.9 ± 2.2 mmHg; *p* = 0.020) without affecting splanchnic blood-flow or systemic hemodynamics. The addition of propranolol to cilofexor additionally reduced splanchnic inflow (−28%) but also mean arterial pressure (−25%) and heart rate (−37%). Conclusion: The non-steroidal FXR agonist cilofexor decreased portal hypertension and reduced liver fibrosis in NASH rats. While cilofexor seems to primarily decrease sinusoidal resistance in cirrhotic portal hypertension, the combination with propranolol additionally reduced mesenteric hyperperfusion.

## 1. Introduction

Non-alcoholic fatty liver disease (NAFLD) is considered the hepatic manifestation of the metabolic syndrome, which affects about one-quarter of the Western population [1]. Approximately 10% of NAFLD patients show a progressive disease course, i.e., non-alcoholic steatohepatitis (NASH) and about 2.5% of NASH patients may develop cirrhosis [1,2]. The prognosis of NASH patients is significantly affected by the degree of hepatic fibrosis and portal hypertension (PH) [3]. PH determines the risk for clinical complications, such as variceal bleeding or ascites. The only oral medication to decrease portal pressure and incidence of PH-associated complications are non-selective beta-blocker (NSBB), like propranolol or carvedilol [4]. However, only 50% of patients achieve a sufficient hemodynamic response to NSBB therapy, and in advanced cirrhosis, NSBB may also have detrimental side effects, such as impairment of renal function or hypotension [5]. While etiological therapy is effective to improve PH, as observed after cure of hepatitis C [6], currently there are no drug therapies specifically approved for NASH. The farnesoid X receptor (FXR) regulates bile acid, lipid and glucose metabolism, inflammation and the sinusoidal vascular tone [7,8]. In preclinical and clinical studies, FXR agonists improved the histological features of NASH and reduced liver fibrosis [9,10]. In a recent phase-2b study, the non-steroidal FXR agonist cilofexor (formerly GS-9674) improved hepatic steatosis and liver transaminases in patients with NASH [11]. Importantly, FXR agonists were also shown to decrease PH in cirrhotic patients and rat models [12,13]. However, there exist no data on the impact of FXR agonism on PH and hepatic hemodynamics in NASH cirrhosis. Thus, we investigated the impact of the non-steroidal FXR agonist cilofexor on PH and liver fibrosis in a rodent model of NASH-associated cirrhosis. We also assessed the hemodynamic effects of a combination of cilofexor with propranolol therapy in NASH-related PH.

## 2. Experimental Section

### 2.1. Ethics

All animal experiments adhered to the ARRIVE (Animal Research: Reporting on In Vivo Experiments) guidelines and were approved by the local Ethics Committee for Animal Research (project identification code: SYN_01_14_FXR_, date of approval: 28.04.2014, Regierungspräsidium Tübingen, and Landesamt/Veterinärsamt Tübingen, Baden-Württemberg, Germany).

### 2.2. NASH Model

NASH was induced in 6–8 week old, male Wistar rats using a choline-deficient high-fat diet (CDHFD, l-amino acid rodent diet with 60 kcal% fat, no added choline and 0.1% methionine, Nr. A06071302, ResearchDiets, New Brunswick, NJ, USA). In order to develop advanced fibrosis, in all NASH rats, 25 mg/kg NaNO_2_ was additionally injected intraperitoneally 3×/week from week 4 onward, according to a published protocol [14]. The last NaNO_2_ injection was administered 3 days prior to the study endpoint. Healthy controls received a standard diet (R/M-H complete feed for rats and mice, V1534, Ssniff^®^, Soest, Germany). All animals were housed on a 12 h light/dark cycle with access to food and water ad libitum in pairs of 2–3 animals per cage on woody litter (Bedding Grade 5, Fibre, H3505-27, Ssniff^®^).

### 2.3. Study Design

**Figure 1:**
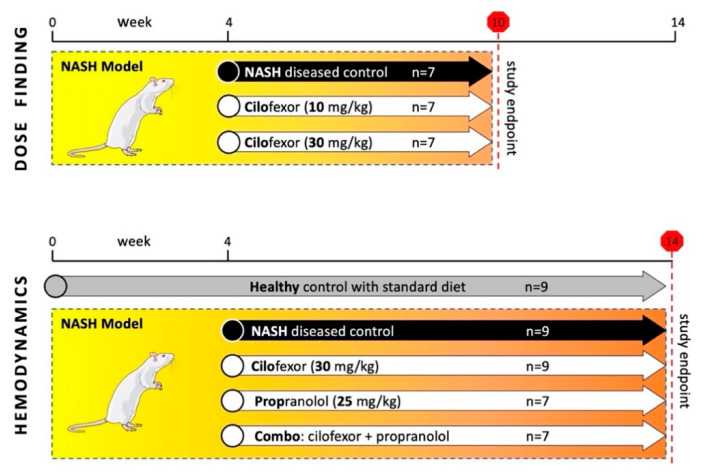
Study design and treatment groups. The NASH model was induced by choline-deficient high-fat diet and injection of NaNO_2_ 3×/week. Initially, a 10-week long dose-finding study was performed with three groups of 7 rats each. The animals received placebo treatment, 10 mg/kg or 30 mg/kg of cilofexor (CILO), respectively, from weeks 4–10. In a subsequent, 14-weeks-long study, four NASH groups (*n* = 7–9) received placebo, 30 mg/kg cilofexor, 25 mg/kg propranolol or a combination of the latter two compounds (Combo) from weeks 4–14 to investigate effects on hepatic hemodynamics.

Our study design comprised two separate animal studies (Figure 1). First, we investigated the effects of cilofexor on liver fibrosis in a dose-finding study using a 10-week NASH model. From week 4 onwards, rats received either 10 or 30 mg/kg of cilofexor. A group of NASH rats without drug treatment (receiving only CDHFD and NaNO_2_ injections) served as NASH-diseased control. Animals were randomized at baseline, and each of the three groups comprised 7 rats. After the 6-week treatment period, animals were euthanized to quantify liver fibrosis content and the hepatic and ileal gene expression. In an independent 14-week NASH model, we subsequently assessed the impact of cilofexor and the NSBB propranolol on systemic and hepatic hemodynamics. From week 4 onward, NASH animals received either 30 mg/kg cilofexor (*n* = 9), 25 mg/kg propranolol (*n* = 7), a combination of both drugs (30 mg/kg cilofexor and 25 mg/kg propranolol, *n* = 7), or no drug treatment (=NASH-diseased control, *n* = 9). A healthy control group (*n* = 9) received standard chow only and no pharmacotherapy. After the 10-week treatment period, hemodynamic measurements were performed, blood was withdrawn and animals were sacrificed for organ harvesting.

### 2.4. Treatment Preparation

The respective test compounds (cilofexor and/or propranolol) were incorporated into the CDHFD by grinding them thoroughly into the pre-warmed diet-mix. The customized drug-containing diet was then pressed into small blocks, dried overnight at room temperature and stored at 4 °C. All diets were used within 5–7 days of preparation. The required test compound concentrations were calculated based on average food intake and rat bodyweight, and doses were adjusted weekly.

### 2.5. Histological Assessment

Paraffin-embedded liver specimens were cut into 5 μm tissue sections and stained using Picro-Sirius red to assess fibrosis. First, sections were incubated for one hour in 0.1% Sirius red F3B in saturated picric acid solution. After rinsing with distilled water, sections underwent staining with Mayer’s hematoxylin, differentiation in 1% HCl and alkalinisation with water. To detect hepatic stellate cells (HSCs) in liver slides, immunohistochemistry was performed using an antibody against desmin (ab32362, Abcam, Cambridge, UK) on the Ventana Discovery ULTRA autostainer platform. Whole slide-scan images of Picro-Sirius red or Desmin stained slides were captured using a Leica AT2 scanner. Quantitative image analysis was performed on the whole slide-scan images using Definiens Architect XD^TM^ and Tissue Studio^®^ software (Definiens AG, München, Germany). The total stained areas were measured and expressed as a percentage of total liver area.

### 2.6. Hepatic Hydroxyproline Content

The hepatic hydroxyproline content was measured using a colorimetric assay (MAK008, Sigma-Aldrich, St. Louis, MO, USA). Briefly, 100 mg of snap-frozen liver tissue was hydrolysed for 6 h at 120 °C in 6 M HCl. Dried samples were oxidized with chloramine-T and incubated in Ehrlich’s perchloric acid solution. Hydroxyproline content was determined photometrically by measuring the absorbance at 560 nm.

### 2.7. Gene Expression Analysis

Gene expression of FXR targets in liver (*shp*, *cyp7a1*, *bsep*) and ileum (*shp*, *fgf15*) samples were performed by reverse-transcription polymerase chain reaction. Briefly, total RNA from 5 mg frozen tissue was isolated using RNAzol RT Reagent (Sigma Aldrich, St Louis, MO, USA) and a RNA Isolation Kit (RNeasy 96 Qiagen, Hilden, Germany) following the manufacturer’s instructions. cDNAs were synthesized from 0.5 μg of total RNA using SuperscriptII™ reverse transcriptase (Life Technologies, Carlsbad, CA, USA) primed with 50 pmol of random hexamers. Quantitative PCR was performed and analyzed using Absolute QPCR Rox Mix (Life Technologies, Carlsbad, CA, USA) and a 384-format ABI 7900HT Sequence Detection System (Applied Biosystems, Foster City, CA, USA). qPCR was conducted at 95 °C for 3 min, followed by 40 cycles of 95 °C for 15 s and 60 °C for 30 s. The relative quantitation of each mRNA was performed using the comparative C_T_ method. Similarly, the hepatic expression of fibrotic (*col1a1*, *pdgfr-β*, *timp1*) genes was assessed. The used primers are summarized in Supplementary Table S1.

### 2.8. Hemodynamic Measurements

Hemodynamic measurements were performed in a blinded manner, under anesthesia (ketamine 100 mg/kg; piritramide 2 mg/kg) and 12 h fasted conditions, as previously described [13]. After cannulation of the femoral artery, mean arterial pressure (MAP) and heart rate (HR) were recorded (catheter PE-50, Smiths Medical, Kent, UK). Portal pressure was invasively measured by advancing a catheter through an ileocolic vein to the portal vein. Splanchnic mesenteric artery blood flow (SMABF) was measured with a non-constrictive perivascular ultrasonic flow-probe (MA1-PRB, Transonic Systems, Ithaca, NY, USA) placed around the superior mesenteric artery (values were normalized to bodyweight). All hemodynamic parameters were continuously recorded with the ML870 PowerLab 8/30 (AD Instruments, Dunedin, New Zealand) and analyzed by LabChart7 Pro software.

### 2.9. Blood Analysis

In the 14-week model, blood samples were analyzed at baseline, week 4, week 9 and at the study endpoint for concentrations of aspartate aminotransferase (AST, U/L) and alanine aminotransferase (ALT, U/L) using a fully automated benchtop analyzer (Respons^®^910, DiaSys Greiner GmbH, Flacht, Germany) with system kits provided by the manufacturer.

### 2.10. Statistics

Results are presented as mean ± standard deviation and group comparisons of parametric data were performed using a two-sided unpaired student’s *t*-test. Primary study outcome parameters were changes of liver fibrosis and portal pressure, respectively. GraphPad PRISM 7 (GraphPad Software Inc, La Jolla, CA, USA) was used for statistical analyses and artwork creation. Two-sided *p*-values < 0.05 denoted statistical significance.

## 3. Results

### 3.1. Cilofexor Reduces Fibrogenesis in NASH in a Dose-Dependent Manner

**Figure 2:**
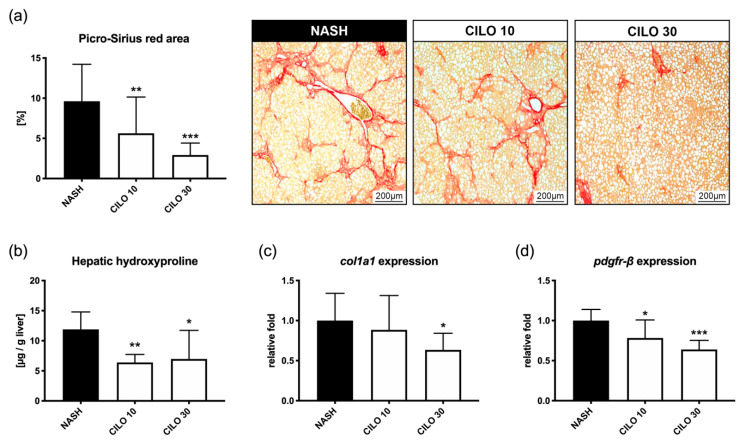
Cilofexor exerts dose-dependent anti-fibrotic properties. (**a**) The anti-fibrotic effects of FXR agonism with cilofexor (CILO) correlate with a dose-dependent decrease in Picro-Sirius red-stained area of liver sections. (**b**) Similarly, hepatic hydroxyproline content was significantly reduced in cilofexor-treated rats. In line with this, expression of (**c**) *col1a1* and (**d**) *pdgf-β* are lowered by FXR agonism. * *p* < 0.05, ** *p* < 0.01, *** *p* < 0.001 vs. NASH (10-week model); two-sided unpaired *t*-test; *n* = 7 per group.

In untreated rats, the 10-week CDHFD/NaNO_2_ NASH model led to severe hepatic steatosis and fibrosis. Compared to these diseased controls (9.62 ± 4.60%), 10 mg/kg (5.64 ± 4.51%, *p* < 0.001) and 30 mg/kg of cilofexor (2.94 ± 1.28%, *p* < 0.001) strongly decreased the Picro-Sirius red-stained area (Figure 2a). In line with this, cilofexor-treated animals presented significantly less hepatic hydroxyproline content (Figure 2b). Moreover, the hepatic expression of profibrogenic genes *col1a1* and *pdgfr-β* was reduced in a dose-dependent manner (Figure 2c,d). These results were mirrored by respective changes in gene expression of hepatic FXR downstream targets *shp* and *cyp7a1*. In ileal tissue, *shp* and *fgf15* expression increased as well, proportionately with the applied cilofexor dose (Supplementary Figure S1). No adverse effects were observed, so the subsequent study was conducted using the higher and more effective cilofexor dose of 30 mg/kg.

### 3.2. Cilofexor Exerts Anti-Fibrotic Mechanisms via Inactivation of HSC

**Figure 3:**
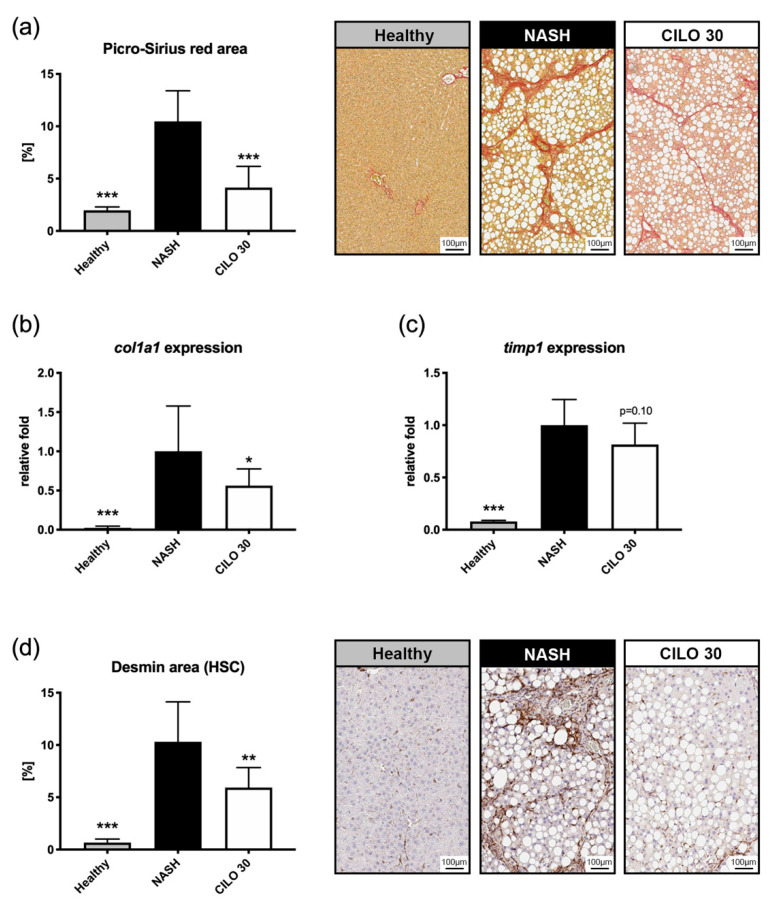
Cilofexor decreases liver fibrosis and hepatic stellate cell activation. (**a**) In the prolonged NASH model, the decrease of Picro-Sirius red area in cilofexor (CILO) treated rats was confirmed. (**b**) In NASH rats receiving cilofexor, hepatic *col1a1* expression was nearly halved by cilofexor treatment and also (**c**) a trend towards reduced *timp1* expression was notable. (**d**) Desmin, a marker of hepatic stellate cell activation, was detected using immunohistochemistry. In cilofexor-treated animals, a significant reduction of desmin area was evident. * *p* < 0.05, ** *p* < 0.01, *** *p* < 0.001 vs. NASH (14-week model); two-sided unpaired *t*-test; *n* = 7–9 per group.

In the extended 14-week NASH model, the beneficial effects of cilofexor on liver fibrosis were confirmatory. Cilofexor significantly reduced the Picro-Sirius red-stained fibrosis area (Figure 3a), which was paralleled by a decrease of hepatic *col1a1* and *timp1* expression (Figure 3b,c). In line with this, we measured strong expression changes of FXR downstream targets in hepatic (*bsep*, *cyp7a1*, *shp*) and ileal (*shp*, *fgf15*) tissue of NASH rats treated with cilofexor (Supplementary Figure S2). To assess activation of HSCs, The desmin area was quantified on histological sections. In NASH rats, hepatic desmin expression was increased 15-fold compared to healthy controls. However, NASH rats receiving cilofexor had 42% less desmin-stained area (Figure 3d).

### 3.3. FXR Agonism Decreases Portal Pressure, Whereas Combination with NSBB Further Decreases Splanchnic Inflow

**Figure 4:**
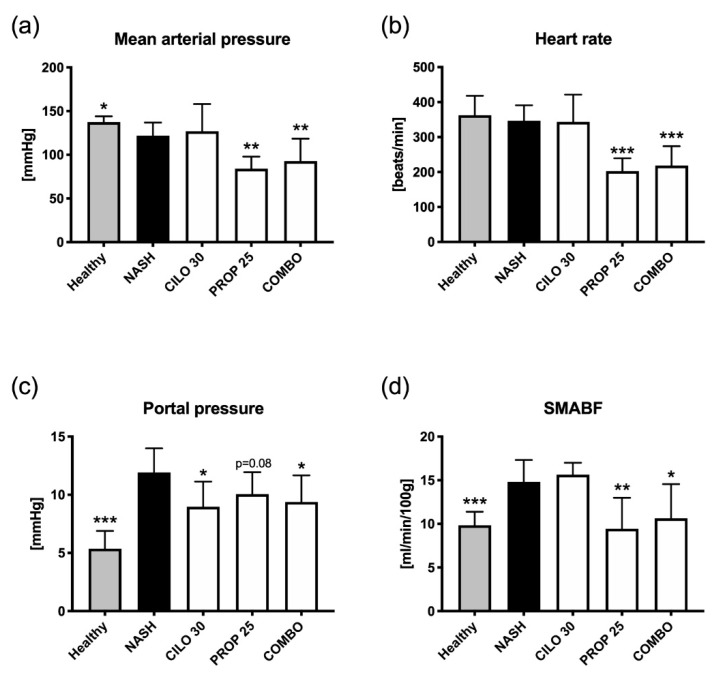
Effects on systemic and hepatic hemodynamics after treatment with cilofexor ± propranolol. (**a**) Mean arterial pressure was decreased in NASH rats and further reduced by treatment with propranolol. Cilofexor (CILO) caused no change in mean arterial pressure. (**b**) Heart rate remained unchanged in NASH animals and also after treatment with cilofexor, whereas propranolol (PROP) decelerated the heart significantly. (**c**) Portal pressure was significantly increased in NASH rats. Cilofexor decreased portal pressure alone and in combination with propranolol (COMBO). However, in NASH rats receiving solely propranolol, only a trend towards decreased portal pressure was observed. (**d**) Superior mesenteric blood flow (SMABF) was increased in NASH animals, and this was not changed by cilofexor. Propranolol treatment reduced SMABF to mean values comparable to the NASH-diseased control group. * *p* < 0.05, ** *p* < 0.01, *** *p* < 0.001 vs. NASH (14-week model); two-sided unpaired *t*-test; *n* = 7–9 per group.

After 14 weeks of NASH, induction rats developed not only liver fibrosis and steatosis, but also portal hypertension. Moreover, NASH animals presented with an elevated SMABF and a decrease in mean arterial pressure, while the heart rate remained stable compared to healthy controls (Figure 4, Supplementary Table S2). NASH rats receiving cilofexor had a 25% decrease in portal pressure, while mean arterial pressure, heart rate and SMABF remained unchanged, as compared to diseased controls. In NASH rats treated with propranolol, we measured a significant decrease in mean arterial pressure and heart rate, a trend to less portal pressure and a strong reduction in SMABF. The combination of cilofexor and propranolol showed a similar decrease in mean arterial pressure and heart rate compared to propranolol alone. However, the combination of cilofexor and propranolol also significantly decreased portal pressure and SMABF in NASH rats.

### 3.4. Neither Cilofexor nor Propranolol Affect Bodyweight or Liver Transaminases

During the 14-week time-course, all animals presented a steady weight gain, and none of the drug treatments had a notable impact on the bodyweight curve compared to the diseased control (Supplementary Figure S3). While in the NASH model, an increase of aspartate (AST) and alanine (ALT) aminotransferase levels was observed, treatment with cilofexor, propranolol or a combination of both did not result in a major change of serum transaminases (Supplementary Figure S4).

## 4. Discussion

In this study using a cirrhotic NASH model with PH, the non-steroidal FXR agonist cilofexor presented significant anti-fibrotic effects and ameliorated PH when administered alone or in combination with propranolol. NASH fibrosis and intrahepatic sinusoidal resistance were reduced via an apparent deactivation of HSCs and inhibition of fibrogenesis.

Research on FXR agonists indicates a therapeutic potential in several etiologies of chronic liver disease. The steroidal FXR agonist obeticholic acid is approved as a second-line treatment for patients with primary biliary cholangitis (PBC) and has also shown anti-fibrotic effects in NASH [10]. Cilofexor is a novel non-steroidal FXR agonist with a favorable pharmacological profile [15] for potential use in NASH and cholestatic disorders. In this cirrhotic rat NASH model, cilofexor was well tolerated with no obvious adverse effects when given in doses up to 30 mg/kg for 10 weeks. The activation of FXR downstream genes was confirmed in hepatic and ileal tissues—supporting target engagement by the agonist cilofexor in both organs.

Cilofexor reduced NASH-associated liver fibrosis in a dose-dependent manner both in terms of histology and gene expression. The anti-fibrotic effects cilofexor reported here resemble those of other FXR agonists in different NASH models [9,16,17]. In this study, we also investigated HSC activation as a key mediator of initiation and progression of liver fibrosis. Desmin was used as an HSC activation marker since its hepatic expression is highly specific for HSCs in rodents [18]. Additionally, we assessed hepatic PDGF receptor expression, which is associated with activated HSCs [19]. Cilofexor treatment was associated with a significant decrease in hepatic desmin staining and also reduced PDGF receptor expression dose-dependently. This is in accordance with previous findings, showing that FXR agonists prevent HSC activation [20] and that cilofexor reduces collagen deposition in HSC/hepatocyte co-cultures [21].

Tissue inhibitor of metalloproteinase-1 (TIMP1) is an important biomarker of matrix remodeling, and TIMP1 levels have been found to be increased in NASH [22]. Moreover, TIMP1 is one of three parameters in the Enhanced Liver Fibrosis (ELF) test, a non-invasive biomarker score to predict advanced fibrosis in patients with NAFLD [23]. In this study, vehicle-treated NASH rats exhibited upregulation of TIMP1, while expression decreased in animals treated with cilofexor. This was also observed in CCl4-injured mice treated with obeticholic acid [20].

To our knowledge, this is the first study to assess PH in response to FXR agonism in a cirrhotic NASH rat model. The CDHFD/NaNO_2_ model was well-suited to assess these effects since it presents a portal hypertensive phenotype with hyperdynamic circulation, whereas pure diet-induced NASH models often only show signs of steatohepatitis and mild fibrosis but lack PH [24]. In this NASH model, treatment with cilofexor significantly decreased portal pressure without altering systemic hemodynamics. While anti-portal hypertensive effects of FXR agonists have been previously observed in rodent models of non-NASH etiology [13,25,26], this is the first experimental study to demonstrate that non-steroidal FXR agonists can reduce portal hypertension in NASH cirrhosis.

Propranolol is a standard treatment for PH, which mainly impacts splanchnic hyperemia but also affects systemic hemodynamics. Accordingly, propranolol treatment reduced portal pressure in NASH rats, but it also strongly decreased mean arterial pressure and heart rate. The combination of FXR agonism and non-selective beta blockade ultimately decreased portal pressure and furthermore SMABF. This is of clinical importance, because high splanchnic flow may increase the risk of variceal bleeding. However, the drug combination did not translate into a synergistic effect on portal pressure reduction—at least in this animal model—and further studies will be necessary to dissect the impact of FXR agonists on the static and dynamic component of PH in a NASH setting.

Contrary to clinical and experimental reports, in our study, the increased level of transaminases remained high in cilofexor-treated rats. This might be attributed to the NaNO_2_ containing NASH model since NaNO_2_ is a proinflammatory stimulus, which was administered throughout the treatment period.

The non-steroidal FXR agonist cilofexor is currently under clinical development for NASH and cholestatic disorders. In fibrotic NASH patients, 12 weeks of cilofexor reduced steatosis, transaminases and TIMP1 levels [27], which was confirmed in a 24-week phase 2 randomized placebo-controlled trial [11]. Preliminary results from the phase 2 ATLAS study (NCT03449446), including NASH patients with advanced (F3–F4) fibrosis, suggest that a low dose of cilofexor did not reduce liver fibrosis. However, a combination of cilofexor with the acetyl-CoA carboxylase inhibitor firsocostat suggested synergistic effects on hepatic fibrosis and NASH activity. In a recent multi-centre study including patients with primary sclerosing cholangitis, cilofexor led to a reduction of alkaline phosphatase, ALT, AST and GGT as well as TIMP1 levels [28] and also improved patient-reported outcomes [29]. A phase 2 trial investigating cilofexor in patients with PBC showed significant reductions in alkaline phosphatase and GGT [30].

Despite the wide use of propranolol in cirrhotic patients [31] and ongoing clinical studies with cilofexor, drug safety needs to be considered, especially when used in combination, or for specific etiologies. In our NASH animal model, liver transaminases and weight development were not altered by any of the drug treatments, compared to the diseased control. However, assessment of drug toxicity was not the main focus of our experiments and potential propranolol- [32] and/or cilofexor-related adverse effects may require further investigations to allow a safe translation into clinical practice.

## 5. Conclusions

In conclusion, the non-steroidal FXR agonist cilofexor decreased portal pressure and reduced liver fibrosis along with HSC deactivation in a rodent NASH model with PH. Cilofexor could be combined with NSBBs, the standard medical treatment for PH, in order to better control mesenteric hyperperfusion. Our study results support further clinical development of the non-steroidal FXR agonist cilofexor for treatment of PH in patients with NASH. Authors should discuss the results and how they can be interpreted in perspective of previous studies and the working hypotheses. The findings and their implications should be discussed in the broadest context possible. Future research directions may also be highlighted.

## Data Availability

The data presented in this study are available on reasonable request from the corresponding author.

## Supplementary Materials

The following are available online at https://www.mdpi.com/2227-9059/9/1/60/s1, Table S1: Sequences of primer used for PCR, Table S2: Summary of hepatic and systemic hemodynamic readouts in the different treatment groups, Figure S1: Gene expression of FXR downstream targets in hepatic and ileal tissue after 6 weeks of cilofexor treatment, Figure S2: Gene expression of FXR downstream targets in hepatic and ileal tissue after 10 weeks of cilofexor treatment, Figure S3: Bodyweight curve of all groups in the 14-week hemodynamic study, Figure S4: Effects of cilofexor, propranolol or the combination of both on serum transaminases in the 14-week hemodynamic study.
