# Supplementary material for: The Non-Steroidal FXR Agonist Cilofexor Improves Portal Hypertension and Reduces Hepatic Fibrosis in a Rat NASH Model
Source: Biomedicines. 2021 Jan 9;9(1):60. doi: 10.3390/biomedicines9010060 (PMC7827357; doi:10.3390/biomedicines9010060)
Supplement: Supplementary file 1 [file biomedicines-09-00060-s001.pdf]

SUPPLEMENTS:

The non-steroidal FXR agonist cilofexor improves portal hypertension and reduces hepatic fibrosis in a rat NASH model

Philipp Schwabl[1,2,3], Eva Hambruch[4], Grant R. Budas[5], Paul Supper[1,2], Michael Burnet[6], John T. Liles[5], Manfred Birkel[4], Ksenia Brusilovskaya[1,2,3], Philipp Königshofer[1,2,3], Markus Peck-Radosavljevic[1,2,7], William J. Watkins[5], Michael Trauner[1], David G. Breckenridge[5], Claus Kremoser[4], Thomas Reiberger[1,2,3,8,9]

[1] *Div. of Gastroenterology and Hepatology, Dept. of Internal Medicine III, Medical University of Vienna, Vienna, Austria.*

[2] *Vienna Hepatic Hemodynamic Lab, HEPEX, Medical University of Vienna, Vienna, Austria.*

[3] *Christian Doppler Lab for Portal Hypertension and Liver Fibrosis, Medical University of Vienna, Vienna, Austria.*

[4] *Phenex Pharmaceuticals AG, Heidelberg, Germany.*

[5] *Gilead Sciences Inc, Foster City, CA, USA.*

[6] *Synovo GmbH, Tübingen, Germany.*

[7] *Department of Internal Medicine and Gastroenterology (IMuG), Hepatology, Endocrinology, Rheumatology, and Nephrology with Centralized Emergency Service (ZAE), Klinikum Klagenfurt am Wörthersee, Klagenfurt, Austria.*

[8] *Ludwig Boltzmann Institute for Rare and Undiagnosed Diseases, Vienna, Austria.*

[9] *CeMM Research Center for Molecular Medicine of the Austrian Academy of Sciences, Vienna, Austria*

TABLE OF CONTENTS:

Supplementary Table S1 2

Supplementary Table S2 3

Supplementary Figure S1 4

Supplementary Figure S2 5

Supplementary Figure S3 6

Supplementary Figure S4 7

Supplementary Table S1 Sequences of primers used for PCR

Gene	Forward Primer	Reverse Primer	Sequence Probe
bsep	AGATACCAGGAAAAGCGTGTG	GCGTAGATGCCAGAGAATTTG	AGCTCCTTCCACCAGAACATGACA
col1a1	CATAAAGGGTCATCGTGGCT	TTGAGTCCGTCTTTGCCAG	TGGTGAACAAGGCCCCTCTGG
cyp7a1	AACGATACACTCTCCACCTTTG	CTGCTTTCATTGCTTCAGGG	TGTTTGCTTGAGATGCCCAGAGGA
enos	ACCTGATCCTAACTTGCCTTG	CAGCCAAACACCAAAGTCATG	TCTTGCCAGAATCCCCGGAAGG
fgf15	GGTTGCTCTGAAGACAATTGC	GTAGCCCAAACAGTCCATTTC	ATCAGCCCGTATATCTTGCCGTCG
pdgfr-β	CAGCAAAATAACAGGACAGCG	GCAATAGCACGAACAGCAAC	CATCAGGAGCCATCTGTAGCCCG
shp	GAGTCTTTCTGGAGCCTTGAG	AGGACTTCACACAATGCCC	AGAGGATAGTGCCTTTCAGGTA TGCGTA
tbp (housekeeper)	CACCAATGACTCCTATGACCC	CAAGTTTACAGCCAAGATTCACG	ACTCCTGCCACACCAGCCTC
timp1	TTTCTGCAACTCGGACCTG	ACAGCGTCGAATCCTTTGAG	TCATCGAGACCACCTTATACCAG CGT

Abbreviations: *bsep*, bile salt export pump; *col1a1*, collagen type I alpha 1; *cyp7a1*, cytochrome P450 family 7 subfamily A; *enos*, endothelial nitric oxide synthase 3; *fgf15*, fibroblast growth factor 15; *pdgfr-β*, platelet-derived growth factor receptor beta; *shp*, small heterodimer partner; *timp1*, TIMP metallopeptidase inhibitor 1; *tbp*, TATA-box binding protein

Supplementary Table S2 Summary of hepatic and systemic hemodynamic readouts in the different treatment groups in the 14-week NASH model setting.

	**Healthy** n=9	**NASH** n=9	**p-value** Healthy vs. NASH	**CILO 30** n=9	**p-value** CILO 30 vs. NASH	**PROP 25** n=7	**p-value** PROP 25 vs. NASH	**COMBO** n=7	**p-value** COMBO vs. NASH
**Heart rate** [bpm]	362±55	346±45	0.521	343±78	0.919	203±36	**<0.001**	218±56	**<0.001**
**Mean arterial pressure** [mmHg]	137±7	122±15	**0.033**	127±31	0.669	84±14	**<0.001**	92±24	**0.009**
**Portal pressure** [mmHg]	5.3±1.5	11.9±2.0	**<0.001**	8.9±2.1	**0.020**	10.1±1.8	0.087	9.4±2.3	**0.033**
**Splanchnic blood flow** [ml/min/100g]	9.8±1.5	14.8±2.5	**<0.001**	15.6±1.3	0.550	9.4±3.5	**0.002**	10.6±3.9	**0.016**

Abbreviations: NASH, non-alcoholic steatohepatitis; CILO 30, 30mg/kg cilofexor; PROP 25, 25mg/kg propranolol, COMBO, combination treatment of cilofexor and propranolol

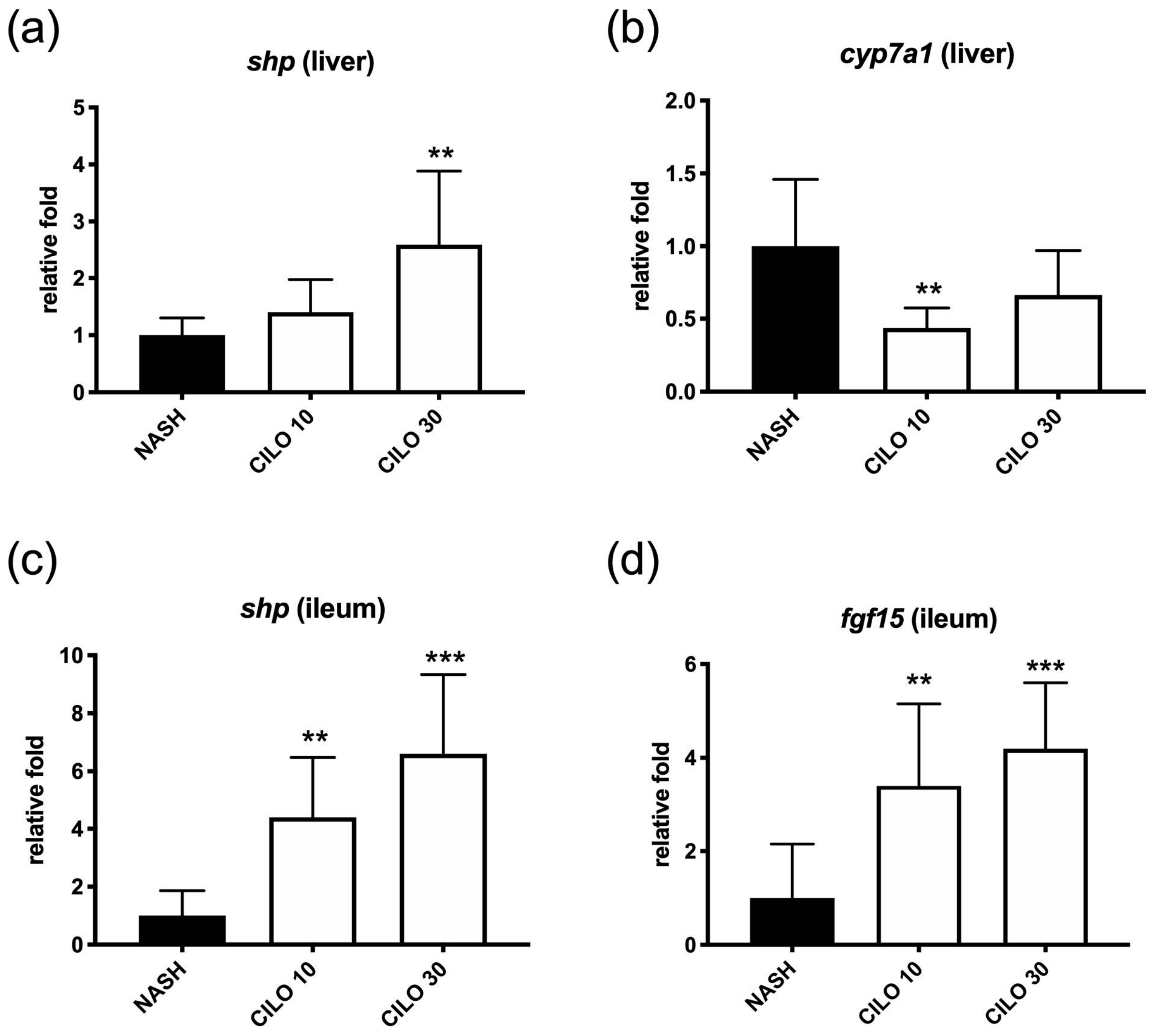

Supplementary Figure S1 Gene expression of FXR downstream targets in hepatic and ileal tissue after 6 weeks of cilofexor treatment. (a) RT-PCR results of *shp* expression in hepatic tissue show a dose dependant decrease in rats treated with cilofexor (CILO). (b) Accordingly, also *cyp7a1* expression is decreased in FXR treated NASH-animals. (c, d) In the ileum, cilofexor upregulated dose-dependently *shp* and *fgf15* expression. *p<0.05, **p<0.01, ***p<0.001 vs. NASH (10-week model); two-sided unpaired t-test; n=7 per group.

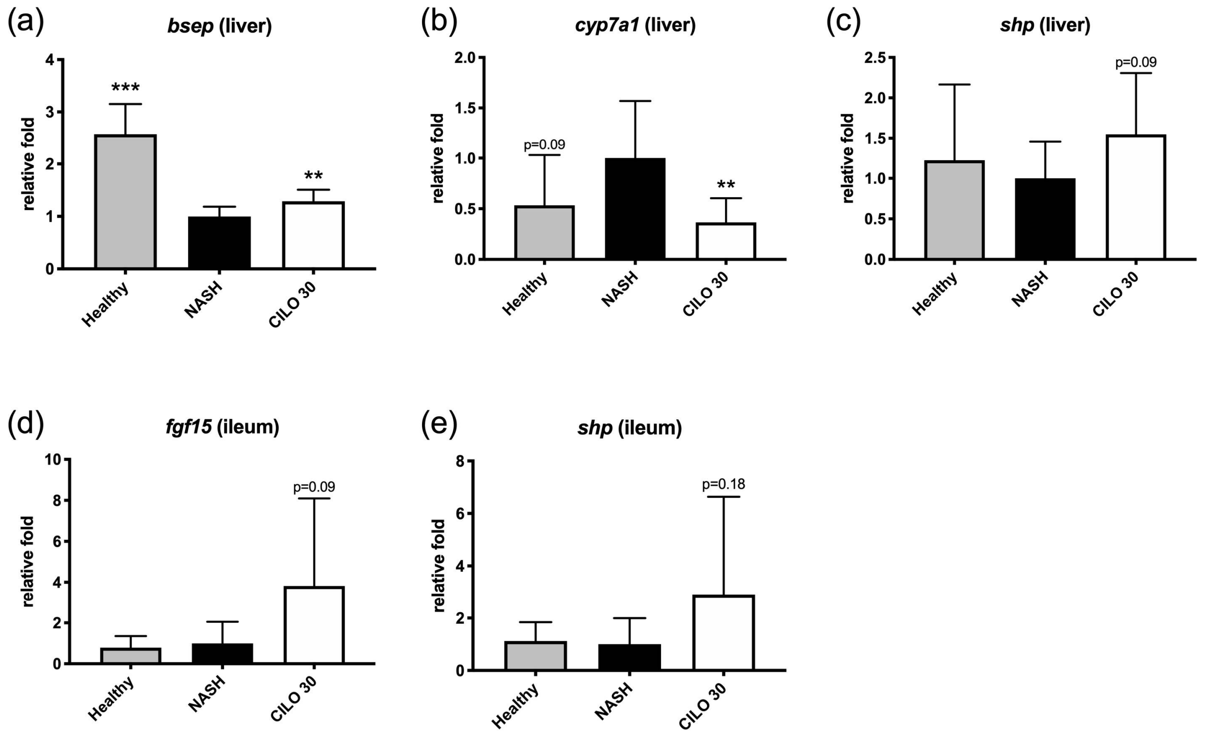

Supplementary Figure S2 Gene expression of FXR downstream targets in hepatic and ileal tissue after 10 weeks of cilofexor treatment. RT-PCRs were performed in (a-c) hepatic and (d-e) ileal tissue to quantify target engagement. (a) Compared to healthy animals, NASH rats presented with significantly downregulated *bsep* expression. In contrast, cilofexor (CILO) treatment upregulated hepatic *bsep* again. (b) Likewise, *cyp7a1* was elevated in NASH animals and declined to normal values by cilofexor. (c) A trend of *shp* upregulation was observed in NASH-cilofexor animals. (d, e) Ileal expression of *fgf15* and *shp* did not change in NASH animals, however cilofexor tended to upregulate both. *p<0.05, **p<0.01, ***p<0.001 vs. NASH (14-week model); two-sided unpaired t-test; n=7-9 per group.

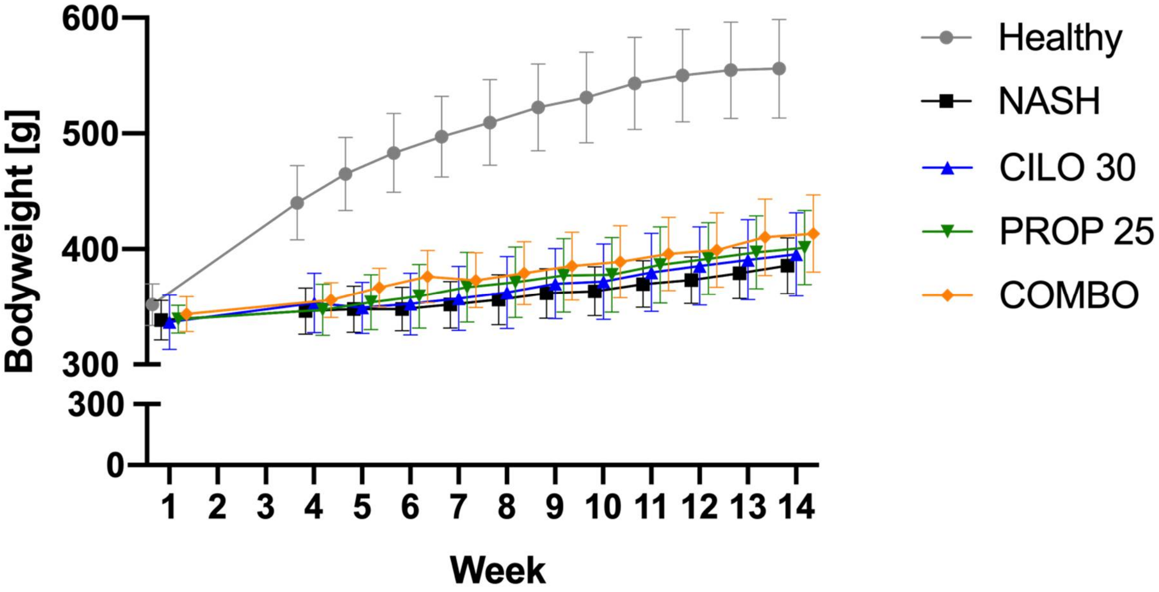

Supplementary Figure S3 Bodyweight curve of all groups in the 14-week hemodynamic study. At baseline all rats presented a similar bodyweight. Healthy animals showed a strong bodyweight gain over the study time-line. In contrast, NASH rats had a slower, yet steady weight gain. None of the drug treatments had a significant impact on body weight.

(a)

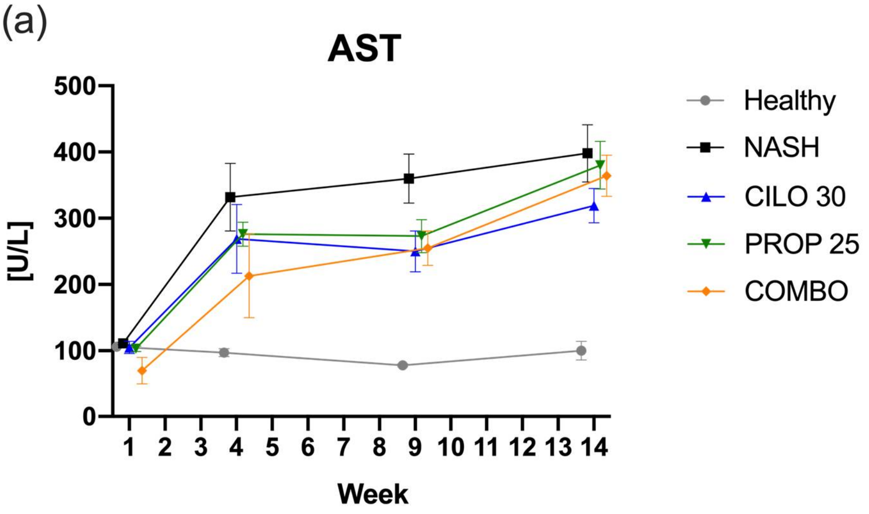

(b)

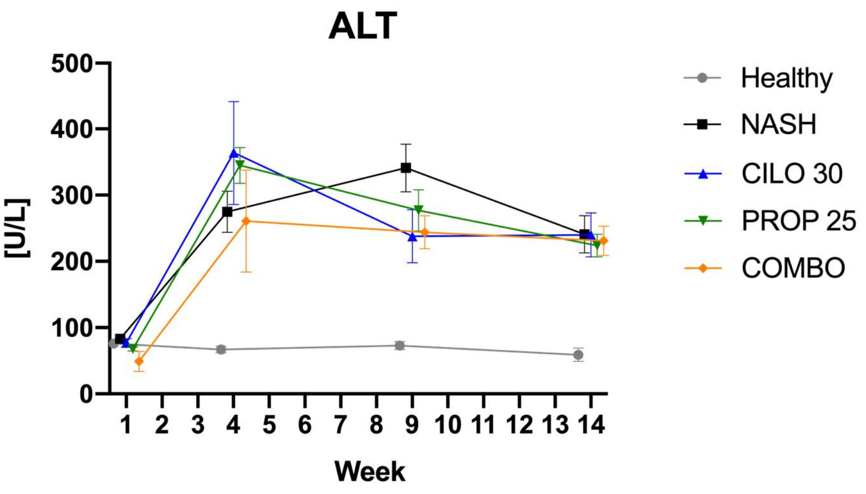

Supplementary Figure S4 Effects of cilofexor, propranolol or the combination of both on serum transaminases in the 14-week hemodynamic study. In NASH animals levels of (a) aspartate transaminase (AST) and (b) alanine transaminase (ALT) were significantly increased, compared to healthy controls. However, at week 14 no changes were observed upon treatment with cilofexor (CILO), propranolol (PROP) or combination of both (COMBO).
